# Parathyroid hormone, vitamin D, and general and abdominal adiposity in bariatric surgery candidates: A cross-sectional analysis of the Tehran Obesity Treatment Study

**DOI:** 10.1097/MD.0000000000051362

**Published:** 2026-10-30

**Authors:** Sepehr Aghajanian, Maryam Barzin, Safa Saleh Nia, Fateme Mohammadifard, Majid Valizadeh, Alireza Khalaj, Amirhesam Alirezaei, Mahmood Bakhtiyari

**Affiliations:** a Student Research Committee, School of Medicine, Alborz University of Medical Sciences, Karaj, Iran; b Neuroscience Research Center, Iran University of Medical Sciences, Tehran, Iran; c Obesity Research Center, Research Institute for Endocrine Sciences, Shahid Beheshti University of Medical Sciences, Tehran, Iran; d Tehran Obesity Treatment Center, Department of Surgery, Faculty of Medicine, Shahed University, Tehran, Iran; e Clinical Research and Development Center, Department of Nephrology, Shahid Modarres Hospital, Shahid Beheshti University of Medical Sciences, Tehran, Iran; f Department of Community Medicine, School of Medicine, Alborz University of Medical Sciences, Karaj, Iran; g Non-communicable Diseases Research Center, Alborz University of Medical Sciences, Karaj, Iran.

**Keywords:** bariatric surgery, calcitriol, mediation, obesity, parathyroid hormone

## Abstract

Obesity is commonly accompanied by low circulating 25-hydroxyvitamin D [25(OH)D], potentially because of volumetric dilution, adipose distribution, and obesity-related changes in 25(OH)D metabolism. Parathyroid hormone (PTH) and 25(OH)D are linked through calcium–phosphate homeostasis and renal conversion of 25(OH)D to calcitriol, but whether 25(OH)D statistically accounts for the association between PTH and adiposity remains uncertain. The data derived from the baseline visit of 4822 participants Tehran Obesity Treatment Study was used to examine the relationship between PTH and 25(OH)D with obesity using regression models. Furthermore, a mediation analysis using potential-outcomes framework was carried out using the aforementioned factors to obtain direct effect of PTH and mediated effect of 25(OH)D on body mass index (BMI) and waist circumference (WC). Higher PTH and lower 25(OH)D were associated with greater adiposity. Compared with the lowest PTH category, PTH > 72.8 pg/mL was associated with 1.89 kg/m^2^ higher BMI, higher odds of morbid obesity (odds ratio: 1.66, 95% confidence interval: 1.33–2.09), and 1.67 cm greater WC. Compared with 25(OH)D < 12 ng/mL, concentrations > 35 ng/mL were associated with 1.81 kg/m^2^ lower BMI, lower odds of morbid obesity (odds ratio: 0.60, 95% confidence interval: 0.48–0.75), and 3.30 cm lower WC. For a PTH increase from 10 to 65 pg/mL, 25(OH)D mediated 7% of the total association with BMI and 9% with WC. Higher PTH and lower 25(OH)D were associated with BMI and WC. Exploratory decomposition suggested that circulating 25(OH)D accounted for only a small portion of the PTH–adiposity association. These findings support careful preoperative mineral-metabolism phenotyping and evaluation of abnormal PTH, but do not establish direction or causality within this framework.

## 1. Introduction

Obesity is a growing global health concern that is influenced by a complex interaction of genetic, metabolic, neuroendocrine, behavioral, psychosocial, and environmental determinants.^[1,2]^ Although chronic positive energy balance is necessary for net fat accumulation,^[3]^ this framework alone does not fully explain the marked interindividual variation in susceptibility to weight gain or the biological mechanisms that determine food intake, energy expenditure, nutrient partitioning, and adipose tissue expansion. Contemporary models of obesity therefore emphasize the integration of peripheral metabolic signals with central neural pathways regulating appetite and energy homeostasis, including signals derived from adipose tissue, the gastrointestinal tract, pancreas, liver, and other organs.^[4]^ In particular, endocrine and metabolic signals that may influence adipose tissue biology but are not routinely incorporated into conventional models of obesity warrant further investigation. Emerging evidence has also suggested that environmental endocrine-disrupting exposures and other metabolic signals may modify pathways involved in energy storage and regulation, although their contribution to human obesity remains under investigation.

Vitamin D and parathyroid hormone (PTH) represent 1 potentially relevant endocrine axis. Despite the widespread nature of inadequate Vitamin D levels in the average global population,^[5]^ obesity by itself is consistently associated with lower circulating concentrations of 25-hydroxyvitamin D [25(OH)D]. Several mechanisms have been proposed, including volumetric dilution, sequestration of this fat-soluble vitamin in adipose tissue, reduced cutaneous exposure to sunlight, and alterations in vitamin D metabolism. Nevertheless, a causal relationship has not been clearly established.^[6,7]^ Serum levels of 25(OH)D serve as established benchmarks for evaluating individuals’ vitamin D status, with deficiency potentially leading to various complications, including secondary hyperparathyroidism and the release of calcium from skeletal tissue, ultimately resulting in bone loss and increased bone fragility. Conversely, numerous studies have demonstrated a notable correlation between obesity in individuals and PTH levels.^[8]^

The literature has highlighted the engagement of PTH and its intricate interplay with vitamin D in the context of obesity, showing that higher PTH and lower 25(OH)D are closely related to truncal fat mass and that PTH does not have a maximal suppression point with regards to vitamin D in male children.^[9,10]^ Serum 25(OH)D is the principal circulating metabolite used to assess vitamin D status and serves as the substrate for subsequent activation to its biologically active metabolite, 1,25-dihydroxyvitamin D; calcitriol.^[11]^ Specifically, reduced ionized calcium stimulates PTH secretion from the parathyroid glands. PTH acts through the PTH type 1 receptor in the renal proximal tubule and stimulates the expression and activity of CYP27B1 (1α-hydroxylase), thereby promoting the conversion of circulating 25(OH)D to calcitriol. Thus, PTH primarily regulates the conversion of 25(OH)D to active vitamin D rather than directly increasing its circulating concentration.

Accordingly, an important unresolved question is whether the observed association between PTH and adiposity is primarily explained by its physiological relationship with 25(OH)D or whether PTH remains associated with adiposity independently of circulating 25(OH)D. The present study therefore investigated the associations of PTH and 25(OH)D with general and central adiposity and explored the extent to which 25(OH)D statistically mediated the association between PTH and body mass index (BMI) and waist circumference (WC).

## 2. Methods

This study was performed within the framework of The Tehran Obesity Treatment Study, a 4-phase, continuous, single-center prospective cohort that began in March 2013 and included participants scheduled for bariatric surgery to assess and compare the effectiveness of different bariatric surgical techniques on metabolic syndrome and other obesity-related comorbidities. Details on the complete set of enrollment and variables and outcomes are available elsewhere.^[12]^ The 4 phases of the studies include preoperative evaluation and baseline data collection; bariatric surgery; short-term postoperative follow-up, including assessments at 1, 3, and 6 months; and long-term follow-up with annual reassessment. Subjects enrolled at the baseline visit were eligible for the current study and served as the basis for the current investigation to examine the association between PTH, 25(OH)D, and obesity (Fig. 1).

**Figure 1.**
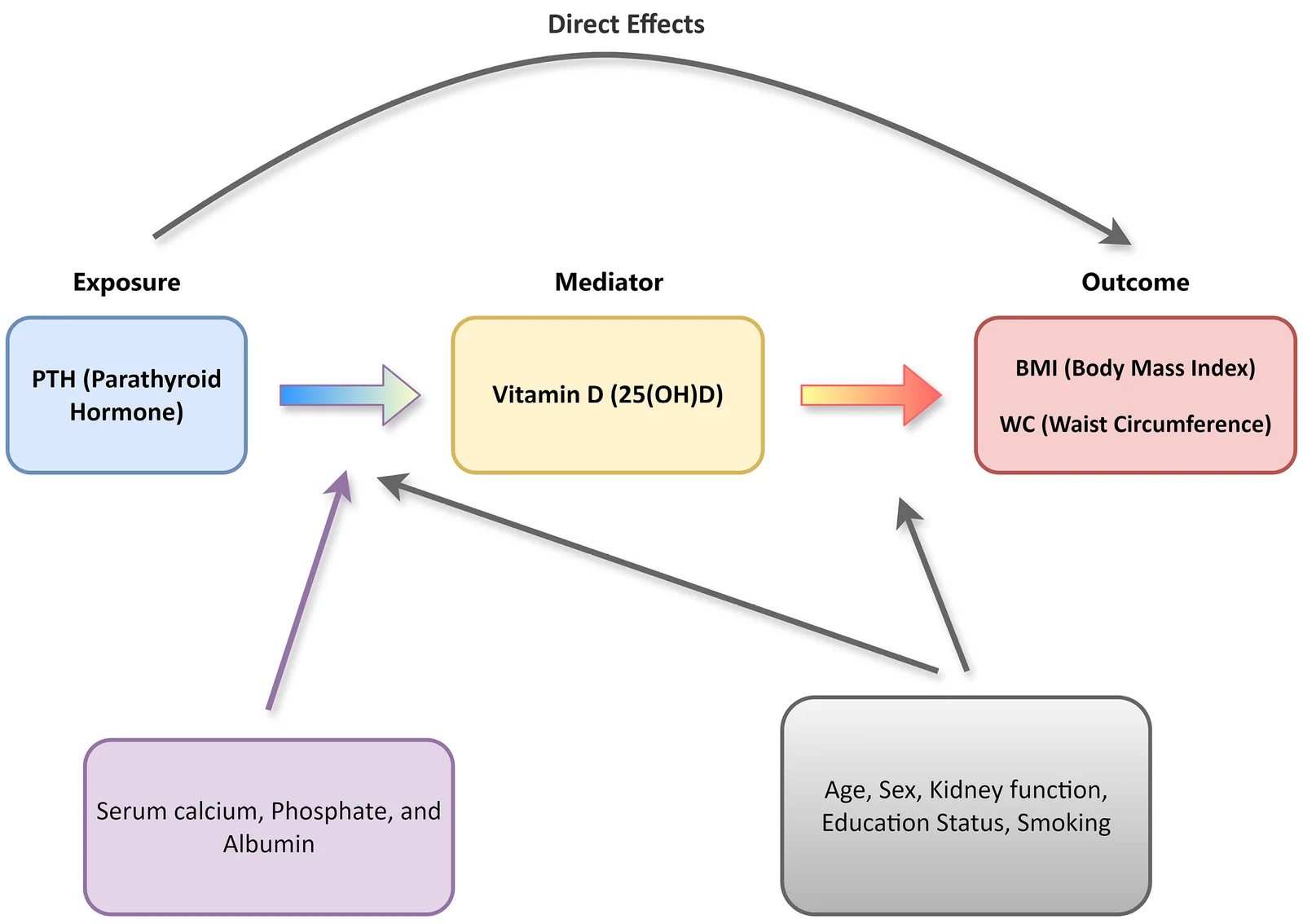
Simplified conceptual framework and causal-directed acyclic graph for the current investigation, showing PTH as the exposure, 25(OH)D as the potential mediator, and obesity measures as the outcome. The arrows for covariates indicate confounding adjustment sets for the relationship between both exposure and mediator, and exposure–mediator and outcome. Calcium, phosphate, and albumin are depicted to illustrate their physiological roles within the PTH–vitamin D axis. Although these variables are involved in mineral-metabolism regulation, they were not treated as conventional confounders of the exposure–mediator–outcome relationship and were, therefore, not included in the primary adjustment set. 25(OH)D = 25-hydroxyvitamin D, BMI = body mass index, PTH = parathyroid hormone, WC = waist circumference.

Other criteria for eligibility included participants aged ≥ 18 years, BMI ≥ 30 kg/m^2^, and those undergoing serum biochemistry and anthropometric assessments. Subjects with missing data on 25(OH)D, PTH, advanced chronic kidney disease (estimated glomerular filtration rate [eGFR] < 60 ml/minute), and missing data on BMI were excluded (Fig. 2).

**Figure 2.**
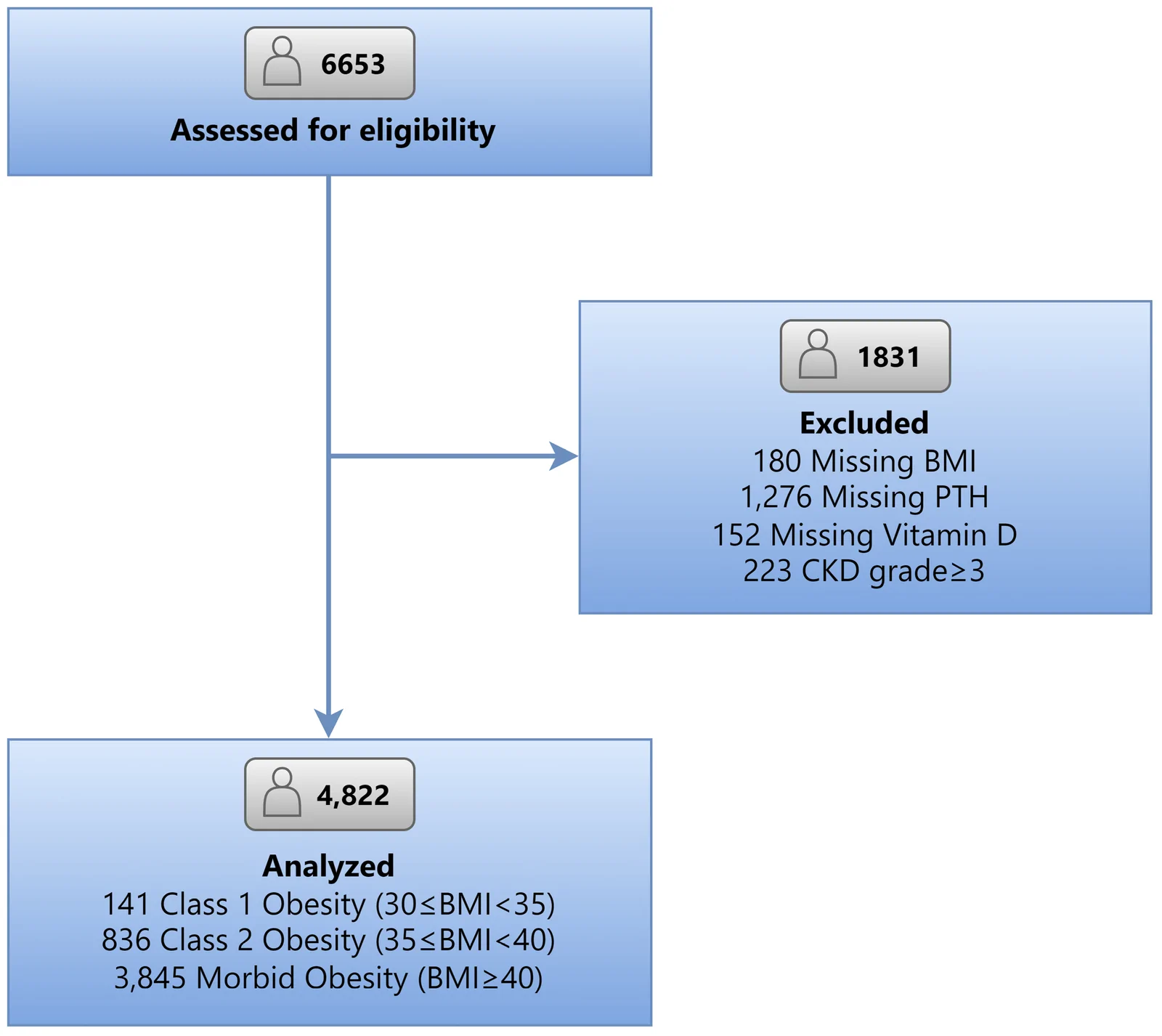
Study flowchart for the inclusion of participants: among 6653 participants with verified data points at baseline, 4822 subjects were included in the study.

Written informed consent was previously obtained from all individuals upon enrollment. This article was derived from the disease registry entitled “Registration of patients in Tehran Obesity Treatment Center” and approved under the ethical code of “IR.SBMU. ENDOCRINE.REC1397.059” (date: 2018-05-08) by the local ethics committee. This work was conducted following the principles of the Declaration of Helsinki.

### 2.1. Measurements and definition of variables

Demographics were obtained for all participants upon enrollment, as elaborated in the study protocol.^[12]^ Educational status was divided into 5 categories, namely, illiterate, those with primary education, secondary education, diploma, and university degrees. Smoking status was trichotomized into never-smokers (< 100 in their entire lifetime), previous smokers, and current smokers. At baseline, venous blood samples were obtained in the morning following an overnight fast of approximately 10 to 14 hours and stored in −80°C ultra-freezers for biochemical assays.

The levels of fasting plasma glucose, serum triglyceride (TG), and total cholesterol were evaluated using an enzymatic colorimetric method with commercial reagents. Simultaneously, high-density lipoprotein-cholesterol (HDL-C) was determined through the precipitation of apolipoprotein B-containing lipoproteins with phosphotungstic acid, utilizing relevant kits. Intact PTH and serum 25(OH)D were measured using electrochemiluminescence immunoassays with Roche Diagnostics reagents on a Roche/Hitachi Cobas e-411 analyzer. Calcium and phosphorus levels were measured using a photometric assay based on the arsenazo III and ultraviolet photometric methods, respectively.^[13]^ The reported intra- and inter-assay coefficients of variation were < 2.6% for iPTH and < 7.5% for 25(OH)D. Regarding fasting plasma glucose, both inter- and intra-assay coefficients of variation at the baseline were determined to be 2.2%. For HDL-C, these coefficients were reported as 2% (inter-assay) and 0.5% (intra-assay). The coefficients for TG were found to be 1.6% (inter-assay) and 0.6% (intra-assay).^[12,14]^

eGFR was calculated based on the 2021 Chronic Kidney Disease Epidemiology Collaboration equation.^[15]^ The original Tehran Obesity Treatment Study definition for diabetes mellitus was used for this study. Dyslipidemia was described as any of the following conditions: serum TGs > 150 mg/dL, total cholesterol > 200 mg/dL, LDL cholesterol > 130 mg/dL, and/or HDL-C < 50 mg/dL in females and 40mg/dL in males.^[16]^ Anthropometric measurements upon the baseline visit were utilized as study outcomes. BMI and WC were selected as measures of general and visceral obesity, respectively. Morbid obesity was defined as WHO class 3 obesity (BMI ≥ 40 kg/m^2^).

### 2.2. Statistical analysis

Continuous variables were summarized using means and standard deviations or medians and interquartile ranges, as appropriate, and categorical variables were summarized as frequencies and percentages. Baseline characteristics were compared between participants with class 1 or class 2 obesity and those with class 3 obesity. To estimate the overall effect of PTH and Vitamin D on obesity, crude linear coefficient and odds ratio (OR) estimates were reported for pentiles of PTH and 25(OH)D for each outcome. The relationship between study exposure, mediator, and BMI was illustrated using locally estimated scatterplot smoothing based on nonparametric local regression models.^[17]^ Vitamin D insufficiency was defined as 25(OH)D < 30 ng/mL and vitamin D deficiency as 25(OH)D < 20 ng/mL. Hypoparathyroidism and hyperparathyroidism status were assigned to those with PTH below 10 pg/mL and higher than 65 pg/mL, respectively.

The final sample contained complete cases for BMI, PTH, and 25(OH)D required for the primary analyses. Other partially missing covariates and WC were handled using multiple imputation by chained equations. Fifty imputed datasets were generated using predictive mean matching for continuous variables and multinomial logistic regression for categorical variables, with 25 burn-in iterations and augmentation for categorical models. The imputation models included sex, PTH, 25(OH)D, BMI, height, weight, age, serum creatinine, eGFR, WC, education, smoking status, calcium, phosphate, and albumin. Variables that were fully observed for the analytic population, including sex, height, weight, BMI, PTH, and 25(OH)D, were retained as regular, non-imputed variables.^[18]^

Mediation analysis was carried out using Stata version 19.5 to calculate direct and indirect effects of the mediator in the framework using Pearl mediation formula. The natural indirect effect (NIE) represents the effect of the exposure transmitted through the mediator when the exposure is held at its higher level, whereas the pure NIE represents the corresponding mediated effect when the exposure is held at its reference level. The total natural direct effect (NDE) represents the direct effect of changing the exposure while the mediator is held at the level it would attain under the higher exposure level. These estimands, together with the NDE, allow the total effect to be decomposed while accounting for potential exposure–mediator interaction.

The mediation models were adjusted for age, sex, eGFR, education, and smoking. As mediation models were estimated separately within each of the 50 imputed datasets, the resulting estimates were combined using Rubin rules. For each parameter, the pooled estimate was calculated as the mean of the estimates across valid imputations, with total variance incorporating both within-imputation and between-imputation variance. Pooled standard errors, 95% confidence interval (CI)s, and hypothesis tests were calculated using Rubin variance and degrees-of-freedom formulae. Alpha was set at 0.05 a priori.

## 3. Results

### 3.1. Study population and baseline characteristics

Among 6653 participants assessed for eligibility, 1831 were excluded because of missing BMI (n = 180), missing PTH measurements (n = 1276), missing 25(OH)D measurements (n = 152), or chronic kidney disease of grade ≥ 3 (n = 223). A total of 4822 participants were therefore included in the final analysis. Of these, 141 had class 1 obesity (BMI 30–35 kg/m^2^), 836 had class 2 obesity (BMI 35–40 kg/m^2^), and 3845 had class 3 (morbid) obesity (BMI ≥ 40 kg/m^2^) (Fig. 2).

The mean age of the study population was 38.9 ± 11.2 years, and 78.3% of participants were female. Educational attainment differed significantly according to obesity category (*P* < .001), with university-level education being more common among participants with class 1 and 2 obesity than among those with class 3 obesity. Overall, 83.0% of participants had dyslipidemia, and 16.5% had diabetes mellitus. Diabetes was more prevalent among participants with class 1 and 2 obesity than among those with class 3 obesity (19.5% vs 15.7%, *P* = .003).

The median PTH concentration was higher among participants with class 3 obesity than among those with class 1 and 2 obesity (49.0 vs 43.2 pg/mL, *P* < .001). Elevated PTH concentrations were present in 27.1% of the overall population and were more frequent among participants with class 3 obesity than among those with class 1 and 2 obesity (28.5% vs 21.3%, *P* < .001). Low PTH concentrations were uncommon and had a similar prevalence in the 2 obesity groups (1.6% in each, *P* = .886).

Median 25(OH)D concentration was lower among participants with class 3 obesity than among those with class 1 and 2 obesity (21.4 vs 23.9 ng/mL, *P* < .001). Vitamin D insufficiency was present in 70.2% of the overall population, with a higher prevalence among participants with class 3 obesity than among those with class 1 and 2 obesity (71.4% vs 65.5%, *P* < .001). Vitamin D deficiency was observed in 43.3% of the overall population and was also more prevalent among participants with class 3 obesity (44.7% vs 37.7%, *P* < .001).

Corrected serum calcium concentrations differed slightly between the 2 obesity categories, although the absolute difference was small (9.08 ± 0.49 vs 9.04 ± 0.47 mg/dL, *P* = .036). Serum phosphate concentrations were similar between groups (*P* = .801), whereas serum albumin concentrations were slightly lower among participants with class 3 obesity (4.37 ± 0.36 vs 4.42 ± 0.37 g/dL, *P* < .001) (Table 1).

**Table 1:** Demographics and baseline characteristics of the included participants.

Characteristics	Number of observations	Total population (n = 4822)	Class 1 and 2 obesity (n = 977)	Morbid obesity (n = 3845)	*P* value
Age (yrs)	4726	38.88 ± 11.15	38.58 ± 10.48	38.96 ± 11.31	.338
Gender	4822				.001
Male		1045 (21.7%)	175 (17.9%)	870 (22.6%)	
Female		3777 (78.3%)	802 (82.1%)	2975 (77.4%)	
Education level	4701				< .001
Illiterate		16 (0.3%)	1 (0.1%)	15 (0.4%)	
Primary education		145 (3.1%)	13 (1.4%)	132 (3.5%)	
Secondary education		480 (10.2%)	43 (4.5%)	437 (11.7%)	
Diploma		1668 (35.5%)	299 (31.1%)	1369 (36.6%)	
University degree		2392 (50.9%)	606 (63.0%)	1786 (47.8%)	
Smoking	4633				.219
Never-smokers		3979 (85.9%)	797 (84.6%)	3182 (86.2%)	
Past smoker		131 (2.8%)	34 (3.6%)	97 (2.6%)	
Current smoker		523 (11.3%)	111 (11.8%)	412 (11.2%)	
Dyslipidemia	4785	3971 (83.0%)	815 (84.1%)	3156 (82.7%)	.299
Diabetes mellitus	4820	793 (16.5%)	191 (19.5%)	602 (15.7%)	.003
PTH (Median [IQR]; pg/mL)	4822	47.80 [33.60–67.0]	43.2 [31.2–61.4]	49.0 [34.40–68.60]	< .001
Hyperparathyroidism		1290 (26.8%)	206 (21.1%)	1084 (28.2%)	< .001
Hypoparathyroidism		57 (1.2%)	12 (1.2%)	45 (1.2%)	.886
25-Hydroxyvitamin D (ng/mL)	4822	22.0 [13.70–32.0]	23.9 [15.60–34.1]	21.4 [13.10–31.30]	< .001
Vitamin D insufficiency		3384 (70.2%)	640 (65.5%)	2744 (71.4%)	< .001
Vitamin D deficiency		2088 (43.3%)	368 (37.7%)	1720 (44.7%)	< .001
Serum corrected calcium (mg/dL)	4260	9.07 ± 0.48	9.04 ± 0.47	9.08 ± 0.49	.036
Serum phosphate (mg/dL)	4686	3.72 ± 0.57	3.71 ± 0.59	3.72 ± 0.57	.801
Serum Albumin (g/dL)	4312	4.38 ± 0.37	4.42 ± 0.37	4.37 ± 0.36	< .001

IQR = interquartile range, PTH = parathyroid hormone.

### 3.2. Association of PTH and vitamin D with adiposity

Increasing PTH concentrations were positively associated with BMI, morbid obesity, and WC, whereas higher 25(OH)D concentrations showed inverse associations with these outcomes (Table 2).

**Table 2:** The association between PTH and vitamin quintile intervals with study outcomes.

Percentile intervals	BMI				Morbid obesity			WC*			
	N	B (95%CI)	*P* value	*P* trend	OR (95% CI)	*P* value	*P* trend	N	B (95% CI)	*P* value	*P* trend
PTH (pg/mL)											
(0–30.7) (Ref.)	968	-	-	< .001	-	-	< .001	608	-	-	.001
(30.7–41.8)	961	0.21 (−0.30–0.78)	.428		1.04 (0.84–1.28)	.735		607	−0.98 (−2.51–0.55)	.208	
(41.8–54.5)	968	0.50 (−0.01–1.01)	.057		1.27 (1.02–1.58)	.029		608	0.35 (−1.18–1.87)	.656	
(54.5–72.8)	961	1.06 (0.55–1.57)	< .001		1.50 (1.20–1.88)	< .001		642	1.33 (−0.18–2.83)	.084	
Above 72.8	964	1.89 (1.38–2.41)	< .001		1.66 (1.33–2.09)	< .001		670	1.67 (0.18–3.16)	.028	
25-Hydroxyvitamin D (ng/mL)											
(0–12) (Ref.)	998	-	-	< .001	-	-	< .001	801	-	-	< .001
(12–18.8)	935	−1.07 (−1.58–−0.56)	< .001		0.78 (0.61–0.98)	.036		624	−0.25 (−1.66–1.17)	.734	
(18.8–25)	985	−1.37 (−1.88–−0.87)	< .001		0.77 (0.61–0.97)	.025		625	−1.17 (−2.59–0.25)	.106	
(25–35)	967	−1.58 (−2.08–−1.07)	< .001		0.64 (0.51–0.81)	< .001		538	−2.19 (−3.67–−0.71)	.004	
Above 35	937	−1.81 (−2.32–−1.29)	< .001		0.60 (0.48–0.75)	< .001		547	−3.30 (−4.78–−1.83)	< .001	

BMI = body mass index, CI = confidence interval, OR = odds ratio, PTH = parathyroid hormone, WC = waist circumference.

* WC percentiles and analyses in this table were carried out in the observed, non-imputed sample.

There was a trend toward higher PTH and lower Vitamin D with BMI (Fig. 3). For instance, for PTH, compared with the lowest concentration category (0–30.7 pg/mL), BMI was 1.89 kg/m^2^ higher among those with concentrations > 72.8 pg/mL (*P* trend < .001). The corresponding OR of morbid obesity was 1.66 (95% CI, 1.33–2.09). A similar pattern was observed for WC, with the highest PTH category associated with a 1.67 cm greater WC compared with the reference category (95% CI, 0.18–3.16; *P* = .028). In contrast, higher 25(OH)D concentrations were associated with lower BMI and lower odds of morbid obesity. Compared with participants with 25(OH)D concentrations of 0 to 12 ng/mL, BMI was lower by 1.07, 1.37, 1.58, and 1.81 kg/m^2^ across the successive concentration categories of 12 to 18.8, 18.8 to 25, 25 to 35, and > 35 ng/mL, respectively (*P* trend < .001). The corresponding ORs for morbid obesity were 0.78 (95% CI, 0.61–0.98), 0.77 (95% CI, 0.61–0.97), 0.64 (95% CI, 0.51–0.81), and 0.60 (95% CI, 0.48–0.75), respectively. The highest 25(OH)D category was also associated with a 3.30 cm lower WC compared with the reference category (95% CI, −4.78–−1.83; *P* < .001). All corresponding trend tests were statistically significant.

**Figure 3.**
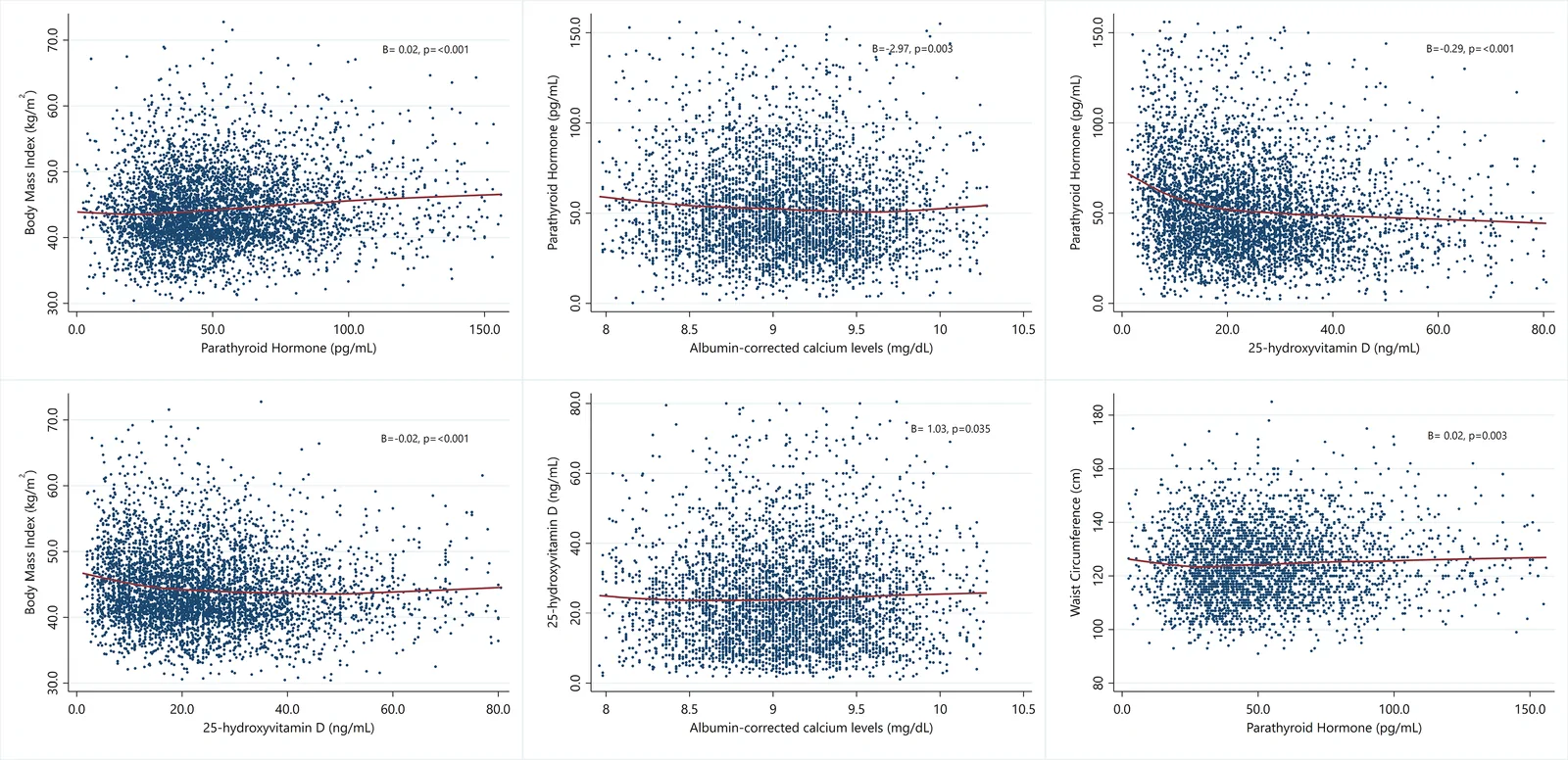
Scatterplots visualizing the pairwise relationship between parathyroid hormone and vitamin D, calcium levels, and obesity indices among study participants. Values above the 99^th^ percentile were clipped for visualization. Higher PTH and lower Vitamin D were associated with higher obesity measurements (Upper and lower left). PTH and vitamin D show a nonlinear inverse relationship with each other (Upper right). The b values were derived from linear univariate regression. PTH = parathyroid hormone.

### 3.3. Mediation analysis

The primary mediation analysis evaluated 25(OH)D as a mediator of the association between PTH and BMI and WC (Table 3). For BMI, a change in PTH from 10 to 65 pg/mL was associated with a total estimated increase of 1.15 kg/m^2^ (95% CI, 0.85–1.45). The NIE mediated through continuous 25(OH)D was 0.08 kg/m^2^ (95% CI, 0.03–0.13), corresponding to approximately 7% of the total effect. The NDE was 1.07 kg/m^2^ (95% CI, 0.76–1.38).

**Table 3:** The results of primary mediation analysis for obesity, parathyroid function status, and vitamin D levels.

Independent variable		Mediator		Outcome	Mediation estimates					
Term	Definition or form	Term	Definition or form		NIE	NDE	PNIE	TNDE	TE	Proportion mediated
Linear increase in BMI										
PTH (10 unit change)	Change from 10 to 65 pg/mL	Vitamin D (10 unit change)	Linear	BMI	*β*: 0.08 [0.03–0.13]	*β*: 1.07 [0.76–1.38]	*β*: 0.05 [−0.02–0.11]	*β*: 1.1 [0.82–1.38]	*β*: 1.15 [0.85–1.45]	0.07 [0.02–0.12]
PTH (10 unit change)	Change from 10 to 65 pg/mL	Vitamin D insufficiency	25-hydroxyvitamin D < 30 ng/mL vs higher	BMI	*β*: 0.05 [0.01–0.09]	*β*: 1.16 [0.89–1.43]	*β*: 0.04 [−0.01–0.09]	*β*: 1.17 [0.91–1.44]	*β*: 1.21 [0.94–1.48]	0.04 [0–0.08]
PTH (10 unit change)	Change from 10 to 65 pg/mL	Vitamin D deficiency	25-hydroxyvitamin D < 20 ng/mL vs higher	BMI	*β*: 0.11 [0.06–0.17]	*β*: 1.15 [0.86–1.44]	*β*: 0.13 [0.06–0.21]	*β*: 1.13 [0.86–1.39]	*β*: 1.26 [0.98–1.54]	0.09 [0.04–0.14]
Hyperparathyroidism	PTH > 65 pg/mL vs 10 ≤ PTH ≤ 65 pg/mL	Vitamin D (10 unit change)	Linear	BMI	*β*: 0.19 [0.07–0.32]	*β*: 1.14 [0.77–1.52]	*β*: 0.07 [0–0.14]	*β*: 1.27 [0.9–1.64]	*β*: 1.34 [0.97–1.71]	0.15 [0.05–0.24]
Hyperparathyroidism	PTH > 65 pg/mL vs 10 ≤ PTH ≤ 65 pg/mL	Vitamin D insufficiency	25-hydroxyvitamin D < 30 ng/mL vs higher	BMI	*β*: 0.11 [0.03–0.19]	*β*: 1.22 [0.85–1.59]	*β*: 0.03 [−0.01–0.08]	*β*: 1.3 [0.93–1.67]	*β*: 1.33 [0.97–1.7]	0.09 [0.02–0.15]
Hyperparathyroidism	PTH > 65 pg/mL vs 10 ≤ PTH ≤ 65 pg/mL	Vitamin D deficiency	25-hydroxyvitamin D < 20 ng/mL vs higher	BMI	*β*: 0.16 [0.05–0.27]	*β*: 1.17 [0.8–1.55]	*β*: 0.14 [0.07–0.21]	*β*: 1.19 [0.82–1.56]	*β*: 1.33 [0.97–1.7]	0.12 [0.03–0.21]
Hyperparathyroidism	PTH > 65 pg/mL vs 10 ≤ PTH ≤ 65 pg/mL	Vitamin D deficiency	25-hydroxyvitamin D < 20 ng/mL vs higher	Morbid obesity (BMI ≥ 40)	OR: 1.05 [1.00–1.10]	OR: 1.48 [1.23–1.77]	OR: 1.04 [1.01–1.07]	OR: 1.50 [1.25–1.79]	OR: 1.55 [1.30–1.85]	0.1 [−0.02–0.21]
Hyperparathyroidism	PTH > 65 pg/mL vs 10 ≤ PTH ≤ 65 pg/mL	Vitamin D insufficiency	25-hydroxyvitamin D < 30 ng/mL vs higher	Morbid obesity (BMI ≥ 40)	OR: 1.05 [1.01–1.10]	OR: 1.47 [1.23–1.76]	OR: 1.02 [1.00–1.04]	OR: 1.52 [1.27–1.81]	OR: 1.55 [1.30–1.85]	0.11 [0.01–0.21]
Hypoparathyroidism*	PTH < 10 vs 10 ≤ PTH ≤ 65 pg/mL	Vitamin D (10 unit change)	Linear	BMI	-	-	-	-	*β*: −0.16 [−1.8–1.47]	-
Linear increase in WC										
PTH (10 unit change)	Change from 10 to 65 pg/mL	Vitamin D (10 unit change)	Linear	WC	*β*: 0.17 [0.05–0.28]	*β*: 1.76 [1.09–2.44]	*β*: 0.21 [0.06–0.36]	*β*: 1.72 [1.1–2.34]	*β*: 1.93 [1.28–2.58]	0.09 [0.02–0.16]
PTH (10 unit change)	Change from 10 to 65 pg/mL	Vitamin D insufficiency	25-hydroxyvitamin D < 30 ng/mL vs higher	WC	*β*: 0.08 [0–0.17]	*β*: 1.86 [1.25–2.46]	*β*: 0.15 [0.02–0.27]	*β*: 1.79 [1.2–2.38]	*β*: 1.94 [1.35–2.53]	0.04 [0–0.09]
PTH (10 unit change)	Change from 10 to 65 pg/mL	Vitamin D deficiency	25-hydroxyvitamin D < 20 ng/mL vs higher	WC	*β*: 0.16 [0.05–0.28]	*β*: 1.95 [1.3–2.61]	*β*: 0.31 [0.13–0.49]	*β*: 1.81 [1.21–2.4]	*β*: 2.12 [1.48–2.75]	0.08 [0.02–0.14]
Hyperparathyroidism	PTH > 65 pg/mL vs 10 ≤ PTH ≤ 65 pg/mL	Vitamin D insufficiency	25-hydroxyvitamin D < 30 ng/mL vs higher	WC	*β*: 0.12 [−0.05–0.29]	*β*: 2.09 [1.24–2.94]	*β*: 0.11 [0.01–0.21]	*β*: 2.1 [1.26–2.94]	*β*: 2.21 [1.37–3.04]	0.05 [−0.03–0.14]
Hyperparathyroidism	PTH > 65 pg/mL vs 10 ≤ PTH ≤ 65 pg/mL	Vitamin D deficiency	25-hydroxyvitamin D < 20 ng/mL vs higher	WC	*β*: 0.17 [−0.07–0.41]	*β*: 2.03 [1.17–2.9]	*β*: 0.27 [0.11–0.42]	*β*: 1.94 [1.09–2.79]	*β*: 2.21 [1.37–3.04]	0.08 [−0.04–0.19]
Hyperparathyroidism	PTH > 65pg/mL vs 10 ≤ PTH ≤ 65 pg/mL	Vitamin D (10 unit change)	Linear	WC	*β*: 0.32 [0.07–0.56]	*β*: 1.9 [1.04–2.76]	*β*: 0.22 [0.07–0.37]	*β*: 2 [1.15–2.85]	*β*: 2.22 [1.38–3.05]	0.14 [0.02–0.27]
Hypoparathyroidism*	PTH < 10 vs 10 ≤ PTH ≤ 65 pg/mL	Vitamin D (10 unit change)	Linear	WC	-	-	-	-	*β*: 0.38 [−3.12–3.89]	-

BMI = body mass index, NDE = natural direct effect, NIE = natural indirect effect, OR = odds ratio, PNIE = pure natural indirect effect, PTH = parathyroid hormone, TE = total effect, TNDE = total natural direct effect, WC = waist circumference.

* Mediation analysis was not carried out due to a lack of association between the variables.

When vitamin D insufficiency (< 30 ng/mL) was used instead of continuous 25(OH)D as the mediator, the corresponding total effect was 1.21 kg/m^2^ (95% CI, 0.94–1.48), with 0.05 kg/m^2^ (95% CI, 0.01–0.09) attributable to the indirect pathway, corresponding to approximately 4% of the total effect. Using vitamin D deficiency (< 20 ng/mL) as the mediator yielded a total effect of 1.26 kg/m^2^ (95% CI, 0.98–1.54), with approximately 9% mediated through the indirect pathway.

Among participants with elevated PTH (> 65 pg/mL), the total estimated effect on BMI was 1.34 kg/m^2^ (95% CI, 0.97–1.71) when continuous 25(OH)D was considered as the mediator. The estimated indirect effect was 0.19 kg/m^2^ (95% CI, 0.07–0.32), corresponding to approximately 15% of the total effect. Using vitamin D insufficiency and deficiency as binary mediators resulted in estimated mediated proportions of approximately 9% and 12%, respectively. No statistically significant association between low PTH and BMI was observed, and mediation analysis was not performed for this subgroup because the prerequisite exposure–outcome association was absent.

For WC, a change in PTH from 10 to 65 pg/mL was associated with a total increase of 1.93 cm (95% CI, 1.28–2.58). The indirect effect through continuous 25(OH)D was 0.17 cm (95% CI, 0.05–0.28), corresponding to approximately 9% of the total effect. Using vitamin D insufficiency or deficiency as the mediator yielded mediated proportions of approximately 4% and 8%, respectively.

Among participants with elevated PTH, the total effect on WC was 2.22 cm (95% CI, 1.38–3.05) when continuous 25(OH)D was used as the mediator. The indirect effect was 0.32 cm (95% CI, 0.07–0.56), corresponding to approximately 14% of the total effect. The corresponding mediated proportions were approximately 5% when vitamin D insufficiency was used as the mediator and 8% when vitamin D deficiency was used.

Overall, the mediation analyses consistently indicated that only a relatively small proportion of the association between PTH and adiposity was statistically attributable to circulating 25(OH)D, while the majority of the estimated association remained in the direct-effect component.

In the sensitivity analyses, the direction of the mediation pathway was reversed, with 25(OH)D treated as the exposure and PTH as the mediator (Table 4). A 10 ng/mL increase in 25(OH)D was associated with a total decrease of 0.48 kg/m^2^ in BMI (95% CI, −0.70–−0.26). The estimated indirect effect through PTH was −0.12 kg/m^2^ (95% CI, −0.18–−0.06), corresponding to 25% of the total effect. For WC, the corresponding total effect was −1.09 cm (95% CI, −1.57–−0.60), with an indirect effect of −0.23 cm (95% CI, −0.36–−0.11), corresponding to 22% of the total effect, suggesting that while PTH values fluctuate largely unrelated to vitamin D levels, vitamin D changes in relation to obesity are more likely to be dependent on PTH; however, given the cross-sectional design, these findings do not establish temporal or causal direction.

**Table 4:** The results of sensitivity mediation analysis using vitamin D as exposure and PTH as the mediating variable.

Independent variable		Mediator		Outcome	Mediation estimates					
Term	Definition or form	Term	Definition or form		NIE	NDE	PNIE	TNDE	TE	Proportion mediated
Vitamin D (10 unit change)	Linear	PTH (10 unit change)	Linear	BMI	*β*: −0.12 [−0.18–−0.06]	*β*: −0.36 [−0.58–−0.14]	*β*: −0.14 [−0.18–−0.1]	*β*: −0.34 [−0.56–−0.12]	*β*: −0.48 [−0.7–−0.26]	0.25 [0.1–0.41]
Vitamin D (10 unit change)	Linear	PTH (10 unit change)	Linear	WC	*β*: −0.23 [−0.36–−0.11]	*β*: −0.85 [−1.34–−0.37]	*β*: −0.21 [−0.29–−0.13]	*β*: −0.88 [−1.37–−0.39]	*β*: −1.09 [−1.57–−0.6]	0.22 [0.07–0.36]
Vitamin D insufficiency	25-hydroxyvitamin D < 30 ng/mL vs higher	PTH (10 unit change)	Linear	BMI	*β*: 0.16 [0.1–0.22]	*β*: 0.49 [0.14–0.85]	*β*: 0.15 [0.07–0.22]	*β*: 0.51 [0.15–0.87]	*β*: 0.65 [0.3–1.01]	0.25 [0.09–0.4]
Vitamin D insufficiency	25-hydroxyvitamin D < 30 ng/mL vs higher	PTH (10 unit change)	Linear	WC	*β*: 0.22 [0.11–0.32]	*β*: 1.12 [0.29–1.95]	*β*: 0.31 [0.14–0.47]	*β*: 1.03 [0.2–1.87]	*β*: 1.34 [0.51–2.17]	0.17 [0.03–0.3]
Vitamin D deficiency	25-hydroxyvitamin D < 20 ng/mL vs higher	PTH (10 unit change)	Linear	BMI	*β*: 0.2 [0.13–0.28]	*β*: 0.86 [0.52–1.19]	*β*: 0.23 [0.15–0.32]	*β*: 0.82 [0.48–1.16]	*β*: 1.06 [0.72–1.39]	0.19 [0.1–0.28]
Vitamin D deficiency	25-hydroxyvitamin D < 20 ng/mL vs higher	PTH (10 unit change)	Linear	WC	*β*: 0.24 [0.09–0.39]	*β*: 1.48 [0.72–2.25]	*β*: 0.45 [0.25–0.64]	*β*: 1.28 [0.51–2.05]	*β*: 1.72 [0.97–2.47]	0.14 [0.03–0.25]

BMI = body mass index, NDE = natural direct effect, NIE = natural indirect effect, PNIE = pure natural indirect effect, PTH = parathyroid hormone, TE = total effect, TNDE = total natural direct effect, WC = waist circumference.

## 4. Discussion

The present study identified consistent associations of higher PTH and lower 25(OH)D concentrations with greater adiposity, including BMI and WC. Across the observed range of PTH concentrations, the magnitude of the association was modest but clinically appreciable: movement from the lower to the upper limit of the reference range was associated with approximately 1.1 kg/m^2^ higher BMI, while participants with PTH concentrations above the reference range had approximately 55% higher odds of class III obesity compared with those within the reference range.

The causality assumptions upon which the current analysis was carried out remain a point of contention. Previous studies have proposed an alternative causal framework in which vitamin D levels were altered secondary to increased deposition in adipose tissue.^[19]^ Based on this hypothetical scenario, PTH rises in response to reduced calcium levels to regulate serum calcium and compensate for hypocalcemia caused by vitamin D deficiency.^[20]^ We challenge this proposed causal network, as total and corrected serum calcium values were not statistically lower in those with morbid obesity. Furthermore, an adjusted analysis with both calcium and inorganic phosphate levels would largely diminish the negative feedback regulation of PTH via vitamin D activity.^[21]^ By excluding cases with impaired renal function, the remaining effect determines the impact of increasing serum PTH levels and its direct effect on increased vitamin D hydroxylation by regulating both 25(OH)D-1 α hydroxylase and 25(OH)D-24-hydroxylase.^[22]^ Nevertheless, our results indicate that the effect of PTH is largely independent of vitamin D, as demonstrated by the low proportion of mediated effect across the total population and study subgroups for both general and abdominal obesity.

Our findings are broadly in line with, but not identical to, previous studies examining PTH, vitamin D, and adiposity. For instance, a number of studies have demonstrated that vitamin D deficiency is associated with metabolic syndrome, impaired insulin secretion, and/or worse glucose tolerance test results in British Asians, which is remediated by vitamin D supplementation.^[23,24]^ Interestingly, in another study on children aged around 9 to 14, vitamin D deficiency was noticeably higher in obese subjects and was associated with higher fat mass index.^[25]^ Contrastingly, while other studies on a severely obese population found a high prevalence of both vitamin D insufficiency and elevated PTH, some report that neither PTH nor 25(OH)D was independently associated with metabolic syndrome after adjustment for age, sex, BMI, and fat mass^[26]^ or that a trend toward a higher rate of metabolic syndrome was only seen in older men with higher PTH.^[27]^ Hjelmesæth et al also showed that among morbidly obese subjects, metabolic syndrome is strongly associated with higher PTH but not 25(OH)D.^[28]^ This association was only seen in women but not men in another study in Korea on participants aged 50 or above,^[29]^ with other adult women studies without age restriction reporting similar findings.^[30]^ Nevertheless, the lack of direct comparability of study outcomes precludes pairwise comparison with the results of our study. The present cohort also represents a particularly severe phenotype of obesity because participants were recruited from a bariatric-surgery program. Consequently, our findings primarily describe the relationship between PTH, 25(OH)D, and variation in adiposity among individuals with established severe obesity rather than the development of obesity in the general population. This distinction is important because vitamin D deficiency and elevated PTH are particularly common in severe obesity, and the relationship between the 2 may be modified by the degree of adiposity, dietary intake, sun exposure, calcium status, and obesity-related alterations in vitamin D distribution and metabolism. A systematic review of bariatric-surgery candidates reported a pooled prevalence of vitamin D deficiency of approximately 59% and deficiency or insufficiency in more than 70% of participants, with substantial geographical variation and comparatively low vitamin D concentrations in Middle Eastern populations.^[31]^

The pathophysiological mechanisms involving this association between Vitamin D and PTH remain obscure. One other proposed pathway involves intracellular calcium signaling in adipocytes. Changes in intracellular calcium concentration can influence lipogenesis, lipolysis, and insulin-regulated glucose metabolism, and experimental literature has suggested that PTH signaling may modify adipocyte calcium handling and promote the antilipolytic effect of phosphodiesterase 3B.^[32]^ The clinical implications of these findings should therefore remain appropriately cautious. Although the association between PTH and adiposity persisted beyond the relatively small statistical contribution of 25(OH)D, our findings do not establish PTH as a therapeutic target for obesity. In particular, clinical trials of vitamin D supplementation in overweight and obese adults have demonstrated that supplementation can suppress PTH and increase circulating 25(OH)D, but evidence that vitamin D supplementation produces meaningful weight-related benefits remains limited.^[33]^ Thus, rather than supporting immediate therapeutic intervention, our findings provide a rationale for further mechanistic and longitudinal studies to determine whether the PTH–adiposity association represents a modifiable pathway and whether interventions targeting the broader PTH–vitamin D axis can influence obesity-related outcomes.

### 4.1. Limitations

The large sample size and availability of standardized biochemical and anthropometric measurements are important strengths of this study. Nevertheless, several limitations should be considered. First, the cross-sectional baseline design prevents determination of temporal sequence and therefore does not establish whether altered PTH contributes to adiposity or represents a consequence of obesity. Second, participants were recruited from a bariatric-surgery population and had severe obesity; consequently, the findings may not be generalizable to individuals with lower BMI or to the general population. Third, although advanced renal dysfunction was excluded, the available dataset did not contain all variables that may be relevant to PTH regulation, including magnesium, fibroblast growth factor 23, 1,25-dihydroxyvitamin D, detailed dietary calcium and vitamin D intake, or comprehensive information regarding vitamin D and calcium supplementation. As such, the outlined limitations underscore the need for cautious interpretation of the findings.

## 5. Conclusions

The findings of this study highlight a substantial correlation between elevated PTH levels and diminished vitamin D levels with abdominal and visceral obesity. Through mediation analysis, it was discerned that the connection between PTH (especially hyperparathyroidism) and obesity is only marginally dependent on vitamin D levels, suggesting that circulating 25(OH)D alone does not explain the observed PTH–adiposity relationship. However, as the cross-sectional design and the absence of several variables involved in calcium–vitamin D–PTH regulation limit causal interpretation, further longitudinal, mechanistic, and interventional studies are required to determine the biological basis and clinical significance of the association. Studies including participants across the full spectrum of BMI, rather than only those with severe obesity, would help determine whether the observed association extends to earlier stages of adiposity and whether it is modified by obesity severity.

## Acknowledgements

The authors would like to thank the Clinical Research Development Unit of Emam Ali Hospital, Alborz University of Medical Sciences, Karaj, Iran for their support, cooperation and assistance throughout the study. The authors would also like to thank the hospital staff, assistants, and coordinators who took part in this research. This article was derived from the disease registry entitled “Registration of patients in Tehran Obesity Treatment Center”

## Author contributions

**Conceptualization:** Alireza Khalaj, Amirhesam Alirezaei, Mahmood Bakhtiyari.

**Formal analysis:** Mahmood Bakhtiyari.

**Methodology:** Sepehr Aghajanian, Mahmood Bakhtiyari.

**Project administration:** Maryam Barzin, Mahmood Bakhtiyari.

**Resources:** Maryam Barzin, Majid Valizadeh.

**Software:** Sepehr Aghajanian.

**Supervision:** Majid Valizadeh, Alireza Khalaj, Amirhesam Alirezaei.

**Validation:** Sepehr Aghajanian, Safa Saleh Nia, Mahmood Bakhtiyari.

**Visualization:** Sepehr Aghajanian.

**Writing – original draft:** Sepehr Aghajanian, Fateme Mohammadifard.

**Writing – review & editing:** Maryam Barzin, Safa Saleh Nia, Mahmood Bakhtiyari.
